# Comparison of mechanical properties of 3D printer resins for occlusal splints using different models of 3D printers

**DOI:** 10.4317/jced.61734

**Published:** 2024-09-01

**Authors:** Arthur Simoes Seidler, Lucas Simino de Melo, João Pedro Justino de Oliveira Limirio, Aldieris Alves Pesqueira, Leandro Augusto Hilgert, Rodrigo Antonio de Medeiros

**Affiliations:** 1 DS, Graduation student, Department of Dentistry, University of Brasilia (UnB), Brasilia, Federal District, Brazil; 2 DDS, postgraduate student at Department of Dentistry, University of Brasilia (UnB), Brasilia, Federal District, Brazil; 3 DDS, MS, postgraduate student at Aracatuba Dental School, Department of Dental Materials and Prosthodontics, Sao Paulo State University (UNESP), Aracatuba, Sao Paulo, Brazil; 4 DDS, MS, PhD, Professor, Department of Dental Materials and Prosthodontics, Sao Paulo State University (UNESP), Aracatuba, Sao Paulo, Brazil; 5 DDS, MS, PhD, Professor, Department of Dentistry, University of Brasilia (UnB), Brasilia, Federal District, Brazil

## Abstract

**Background:**

Considering the development of new 3D printing technologies that use different printing techniques, further studies must be conducted to evaluate the impact of different printing systems on the mechanical properties of 3D-printed materials. This study aimed to evaluate the mechanical properties of 3D-printed materials for occlusal devices using different 3D printers and printing layer thicknesses.

**Material and Methods:**

Ninety rectangular samples were manufactured and divided into nine groups according to the 3D printer model they were printed on (AnyCubic Mono X, Elegoo Mars 2, or FlashForge Hunter) and the layer thickness (20, 50, or 100 µm) and were subjected to superficial microhardness, flexural resistance, and elasticity modulus tests. The results were analyzed using two-way analysis of variance and Tukey’s statistical tests, with a significance level of 5%.

**Results:**

The type of 3D printer significantly affected superficial microhardness (*p* = 0.007). Flexural strength showed a significant interaction between the 3D printer and layer thickness (*p* = 0.005), with both factors independently influencing flexural strength (printer: *p*< 0.001, layer thickness: *p*< 0.001). Elasticity modulus was significantly influenced by the 3D printer type (*p*< 0.001) and the interaction between both factors (*p* = 0.004). The AnyCubic Mono X 3D printer with a 20 µm layer thickness exhibited more consistent mechanical properties than the other printers.

**Conclusions:**

Variations in printing systems and layer thicknesses can impact the mechanical properties of 3D-printed materials.

** Key words:**CAD-CAM. Bruxism. Temporomandibular disorders. Mechanical tests; 3-D printing.Care Team.

## Introduction

Temporomandibular dysfunction (TMD) describes functional problems related to the temporomandibular joint and correlated structures, such as masticatory muscles.(1,2) Among many etiological factors, we can describe environmental, biological, psychological, biomechanical, and neuromuscular factors that could play a major role or function concurrently in the development of TMD (1,3). The most prevalent symptoms in patients diagnosed with TMD include, but are not limited to, lower jaw pain, articular pain, toothache (with non-dental origins), earache, headache, and functional limitation of the lower jaw (4,5).

The factors analyzed to the etiology of TMD, following the psychosocial model, are biological factors (genetic or biochemical predisposition factors), psychological factors (anxiety, stress, depression, among other factors), and social factors (culture, familiar behavior, socioeconomical conditions, among other factors) (3). Another factor that could contribute to developing TMD is bruxism (6). Bruxism is the parafunctional activity of masticatory muscles regulated by the central nervous system (7), occurring during sleep or awakening, in which repetitive or sustained dental contact is associated with the static or dynamic contraction of the mandible (8).

One treatment method for TMDs and the deleterious effects of sleep bruxism is the use of occlusal devices (9). Occlusal devices are commonly manufactured with acrylic resins such as polymethylmethacrylate (PMMA). Recently, new methods for the production of occlusal devices have been developed, including computer-aided design and computer-aided manufacturing (CAD/CAM) technologies. This method consists of projecting, fabricating, and materializing devices using computers and additive manufacturing (3D printers) (10) or regressive manufacturing (milling units for PMMA plates or blocks). Recent studies have shown that modern production methods are efficient in manufacturing occlusal devices (11).

The production of occlusal devices through additive manufacturing is of great interest for dental clinical practice because of its fast processing and diminishing need for dental laboratories. With the rapid development of different 3D printing technologies, there is a need for studies that evaluate different printing techniques that could influence the indication of clinical practice and their impact on the final product.

Various studies have shown that, regarding project trueness and dimension accuracy, different types of 3D printers present similar results (12,13). However, there is still a lack of studies comparing the mechanical properties of the materials for the production of occlusal devices used for 3D printing within these different printers. There are two types of printers, DLP (Digital Light Processing) and MSLA (Mask Stereo Lithography Apparatus). DLP is a 3D-printing technology which consists in the emission of an image by a high precision projector reflected by a mirror; MSLA, in the other hand, uses a sequence of LEDs that emit light on an LCD screen. On both printers, the user can customize various printing settings, which may affect directly on the quality, precision and printing time, such as the layer thickness. The layer thickness is measured in microns and refers to the height of each layer that is sintered during the 3D printing process. Larger layer heights, such as 100 µm, reduce printing time but may affect the effective polymerization of the resin. Meanwhile, smaller layer heights, like 20 µm, increase printing time, potentially impacting clinical use.

This study aimed to compare the mechanical properties of occlusal device resins for 3D printing by varying the 3D printer and using different layer thicknesses for 3D printing. The research hypothesis for this study was that different printers and layer thicknesses can influence the mechanical properties of the resin used for printing occlusal devices on 3D printers.

## Material and Methods

-Samples manufacture

The rectangular samples, with dimensions of 64.3x10.3x3.3 mm, were printed using the following 3D printers: FlashForge Hunter, DLP with FHD 1080p resolution projector (Flashforge, Sao Jose dos Campos, Sao Paulo, Brazil), AnyCubic Mono X, MSLA monochromatic 4 K with 3840x2400 pixel density (Anycubic Technology Company, Hongkong, Hongkong China), and Elegoo Mars 2, MSLA monochromatic 2 K with 1620x2560 pixel density (Elegoo, Shenzhen, Guangdong, China), all of this printer emitting UV light at the wavelength of 405nm, as of the recommendation of the resin manufacturer.

The additive manufacture of the samples was made of 3D printer resin, specific for the production of occlusal devices (Prizma 3D Bio Splint; Makertech, Sao Cristovao, Sao Paulo, Brazil) and used for all 3D printers. The settings for exposure time were set following the manufacturer’s recommendations and calibrated with a calibration test, the test being a 15x20 mm rectangle, that was repeatedly printed with small variations in the printer settings, until there was no difference between the dimensions set on the digital project and the printed parts.  Table 1 lists the obtained values. Post-processing and curing of the samples were performed by washing the samples with isopropyl alcohol for 5 min and 30 s in a Wash and Cure unit (Anycubic Technology Company, Hongkong, Hongkong, China) that cleans the excesses resin circulating the isopropyl alcohol and curing them in an ultraviolet (UV) curing station (405 nm) for 5 min.

The FlashForge Hunter printer displays an additional setting compared to the others, that is, light intensity. This parameter was set to 40% according to the resin manufacturer.

Ninety samples were manufactured and divided equally on groups, according to the printer and layer thickness used on the production (20, 50, or 100 µm).

• Group 1 (G1) – Mono X – Layer thickness 20 µm (n=10)

• Group 2 (G2) – Mono X – Layer thickness 50 µm (n=10)

• Group 3 (G3) – Mono X – Layer thickness 100 µm (n=10)

• Group 4 (G4) – Mars 2 – Layer thickness 20 µm (n=10)

• Group 5 (G5) – Mars 2 – Layer thickness 50 µm (n=10)

• Group 6 (G6) – Mars 2 – Layer thickness 100 µm (n=10)

• Group 7 (G7) – Hunter – Layer thickness 20 µm (n=10)

• Group 8 (G8) – Hunter – Layer thickness 50 µm (n=10)

• Group 9 (G9) – Hunter – Layer thickness 100 µm (n=10)

The samples were polished with standardized #200, #600, and #1000 grit sandpaper disks (Carbarnet; Buehler, Lake Bluff, Illinois, United States) and #800 and #1200 grit sandpaper disks (Microcut; Buehler, Lake Bluff, Illinois, United States), from high to low abrasion, attached to an automated polishing machine (AutoMet 250; Buehler, Lake Bluff, Illinois, United States) under constant water irrigation and rotation at 300 rpm for 30 s per surface. Subsequently, the samples were finished using a polycrystalline diamond solution (MetaDi Supreme; Buehler, Lake Bluff, Illinois, United States) on each surface.

-Superficial Knoop microhardness test

Knoop microhardness values were determined using a Microhardness Tester (HMV-2T, Shimadzu Corporation, Barueri, Sao Paulo, Brazil) with a load of 25 g for 10 s in the last printed surface. The analysis measurement spots of the specimen were made on the center and near each border. The mean of the three spot measurements was registered.

-Flexural strength and modulus of elasticity

For the three-point flexural strength test and modulus of elasticity, each sample was placed on a 50 mm-long support for the three-point flexural test. A vertical force was applied at the mid-point of the specimen on a universal testing machine EMIC model DL 3000 (EMIC, Sao Jose dos Pinhais, Sao Paulo, Brazil) at a constant speed of 5 mm/min until fracture.(14) Equations for calculations were performed as Tijana *et al*.(15)

-Statistical analysis

Two-way analysis of variance (ANOVA) was performed to assess the statistical differences between groups for superficial Knoop microhardness, flexural strength, and modulus of elasticity. Tukey’s test was used to compare groups with statistical differences. All statistical analyses were performed using the statistical software SPSS Statistics 17.0 (SPSS Inc.). *P* < 0.05 indicated statistical significance.

## Results

The results of the two-way ANOVA are presented in Supplementary material 1 (Superficial Knoop microhardness), Supplementary material 2 (flexural strength), and Supplementary material 3 (modulus of elasticity).

Two-way ANOVA demonstrated that only the type of 3D printer had a significant main effect (*p* = 0.007) on superficial Knoop microhardness (Supplement 1) (http://www.medicinaoral.com/medoralfree01/aop/jced_61734_s01.pdf). After performing the Tukey test ( Table 2) for superficial Knoop microhardness, we noticed that there was no statistical difference between printers for the 20 µm layer thickness. For 50 µm, the Mono X 3D printer showed higher results, and for 100 µm, the Hunter 3D printer showed lower results. When comparing different layer thicknesses on the same printer, the Hunter 3D printer showed lower values for 100 µm, and for the Mars 2 and Mono X 3D printers, the compared layer thicknesses did not differ significantly.

Two-way ANOVA showed that the interaction between the 3D printer and layer thickness was significant (*p* = 0.005). Both main factors, printer (*p* < 0.001) and layer thickness (*p* < 0.001), significantly affected flexural strength. For flexural strength, after performing the Tukey test ( Table 2), we noticed that there was no statistical difference between the printers for the 20 µm layer thickness. For 50 and 100 µm, Mono X showed higher values than those of the remaining 3D printers. When comparing the 20 µm layer thickness within the same printer, Hunter and Mars 2 showed higher values.

Two-way ANOVA revealed that the interaction between 3D printer and layer thickness was significant (*p* = 0.004), with the type of 3D printer being the only main factor that had a significant effect (*p* < 0.001) on elasticity modulus. For modulus of elasticity, after performing the Tukey test ( Table 2), we noticed that there was statistical difference between the printers for the 20 µm layer thickness. In this analysis, Mars 2 showed higher mean values when compared to Hunter and Mono X. For the 50 µm, Mono X showed lower values, and for the 100µm layer thickness, Hunter showed lower values than those of the remaining 3D printers. When comparing the 50 µm layer thickness within the same printer, Hunter showed higher values than those of the remaining systems. For Mars 2 and Mono X, the layer thicknesses did not differ significantly between the groups.

## Discussion

Statistical differences were observed between the 3D printers and the layer thicknesses, validating the research hypothesis of the present study.

Regarding the layer thickness, the results showed that overall, the mechanical properties were better at 20 µm. This is supported by the fact that thinner layers receive more energy, and therefore, proportionally, a higher monomer-to-polymer conversion (12). The only test in which the values remained the same despite the higher layer thickness was the superficial Knoop microhardness (except for Hunter 100 µm). Microhardness seems to be more strongly associated with the material used (9) and post-impression procedures after curing, such as finishing and polishing (16). Hunter might have had a worse performance on 100 µm due it provided less energy, which will be discussed further, and the machine used to measure microhardness penetrated until the less polymerized layers, lessening its statistical value.

However, in this study, Hunter presented worse overall mechanical properties than those of the remaining two 3D printing systems when 100-µm thick-layered samples were printed. This might have been related to the power delivered by the printer. The light intensity recommended by the manufacturer is 40%; it is possible that, because the layer is thicker, the layers more distant to the light source might not have achieved the same monomer-polymer conversion than the layers closer to the light source, diminishing the mechanical properties (16). Possibly, if a new test was to be performed with a higher light intensity for Hunter 3D printer, the results found on 100µm could be closer to the results found on other printers. Further studies are needed to test this hypothesis.

In this regard, the difficulty of polymerization of thicker layers and the interference of light intensity can explain why Mono X was the only printer that maintained a great printing standard among different layer thicknesses. Considering that Mono X has the most potent light source, or a LED array with the higher nominal power, (Hunter has a similar light source, but the samples were printed using only 40% of its maximum power due to the recommendation of the resin manufacturer), more regular polymerization of different layer thicknesses was obtained. The higher conversion of monomers might have positively influenced the results presented by this printer when different layer thicknesses were used (9).

Notably, different resins and materials used for 3D printing present different results. The resin used in this study is recommended by the manufacturer for preparing occlusal devices, but other resins for other purposes may provide different results, such as resins for temporary or definitive restorations (17).

The choice of printer type and layer height will be influenced by the therapeutic objective. Considering the results obtained in this study, patients with severe bruxism are eligible to use 3D printed devices printed with a 20-µm layer thickness on 3D printers with the most powerful light sources, such as Mono X. If a device is indicated for painful TMD or protection of crowns in patients who are not diagnosed with bruxism, we can indicate the use of devices printed at 20 or 50 µm, selecting printers with powerful light sources. To ensure the laboratory technician can produce an occlusal device with the optimal layer height and printer, the dentist needs to provide information about the diagnosis, specifically, whether the patient is experiencing severe bruxism and/or painful temporomandibular disorder. Although the results revealed statistical variances in mechanical properties concerning layer height and printer type, it remains unclear from the present study whether these differences hold clinical significance. Randomized clinical studies are required to validate these findings. Randomized clinical trials must be performed to confirm these findings.

The limitations of this study include: the use of a single brand of 3D printer resin, considering the results could be influenced by the problems and benefits of the chosen resin. Moreover, important methodologies were not performed, such as the degree of conversion, scanning electron microscopy analyses, and color stability. More studies should be conducted to verify our results and to better comprehend the clinical applications and indications for each patient.

## Conclusions

The mechanical properties of the resins used for the 3D printing of occlusal devices are influenced by the 3D printer model, and by the layer thickness used for printing. The Mono X 3D printer and a 20-µm layer thickness presenting the most consistent and higher strength results respectably among the parameters and printers analyzed.

## Figures and Tables

**Table 1:** Exposure time for each layer by printer and by thickness.

	Mono X	Hunter	Mars 2
20 µm	22 s for the eight bottom layers (first printed) and 1.5 s for other layers	15 s for the eight bottom layers (first printed) and 2.5 s for the other layers	20 s for the eight bottom layers (first printed) and 2 s for the other layers
50 µm	30 s for the eight bottom layers (first printed) and 2.5 s for other layers	20 s for the eight bottom layers (first printed) and 4 s for the other layers	30 s for the eight bottom layers (first printed) and 3 s for the other layers
100 µm	40 s for the eight bottom layers (first printed) and 5 s for the other layers	35 s for the eight bottom layers (first printed) and 4.5 s for the other layers	35 s for the eight bottom layers (first printed) and 5 s for the other layers

**Table 2:** Tukey test for superficial Knoop microhardness, flexural strength and elasticity modulus.

Tests	3D Printer models	Layer thickness		
		20µm	50µm	100µm
Knoop hardness (kgf/mm^2^)	Hunter	12.61 (1.83) Aa	11.4 (2.04) Aa	10.97 (1.26) Ab
	Mars 2	12.14 (2.2) Aa	12.59 (1.5) Aa	13.08 (1.65) Ba
	Mono X	12.65 (1.74) Aa	13.44 (1.31) Ba	13.06 (1.3) Ba
Flexural strength (MPa)	Hunter	83.66 (22.62) Aa	68.77 (15.49) Ab	45.66 (15.63) Ac
	Mars 2	86.91 (18.74) Aa	59.49 (14.79) Ab	48.42 (4.80) Ab
	Mono X	95.63 (8.59) Aa	95.35 (5.93) Ba	85.16 (10.55) Ba
Flexural modulus (MPa)	Hunter	1378.12 (182.10) Aa	1481.03 (175.11) Aa	1128.04 (286.87) Ab
	Mars 2	1581.96 (239.36) Ba	1465.01 (177.37) Aab	1397.58 (247.16) Bb
	Mono X	1211.24 (204.98) Aa	1198.45 (170) Ba	1321.11 (109.43) Ba

## Data Availability

The datasets used and/or analyzed during the current study are available from the corresponding author.
